# Rifampicin-Associated Secondary Minimal Change Disease Presenting with Nephrotic Syndrome in a Pulmonary Tuberculosis Patient

**DOI:** 10.1155/2021/5546942

**Published:** 2021-04-14

**Authors:** Satyanand Sathi, Anil Kumar Garg, Manoj Kumar Singh, Virendra Singh Saini, Devinder Vohra

**Affiliations:** Department of Medicine, S.M.M.H. Government Medical College, Saharanpur, Uttar Pradesh, India

## Abstract

Various extraglomerular disease processes have been associated with drug-induced secondary minimal change disease (MCD). In a majority of cases, preferably, a hypersensitivity reaction appears to be involved, and in some cases, there is direct toxic effect over glomerular capillaries. There are several reports to demonstrate that rifampicin has been associated with various nephrotoxic adverse effects, but rifampicin-induced secondary minimal change disease (MCD) is very rare. Here, we report the case of a young adult male who presented with nephrotic proteinuria with bland urine sediment after one month of initiation of rifampicin treatment for pulmonary tuberculosis. The patient had no proteinuria before the start of antituberculosis treatment. Renal biopsy showed nonproliferative glomerulopathy and immunofluorescence did not show significant glomerular immune deposits. Electron microscopy showed diffuse effacement of visceral epithelial cell foot processes and did not show any presence of glomerular immune complexes and thickening of glomerular basement membrane, promoting the diagnosis of minimal change nephrotic syndrome. The patient got complete remission after discontinuation of rifampicin.

## 1. Introduction

Primary minimal change disease (MCD) is characterized by a disorder of charge selective glomerular permselectivity and typical morphological changes in glomerular capillaries without well-defined extra-glomerular disease process [1]. In secondary MCD, the characteristic changes in glomerular permselectivity and morphology are elicited, directly or indirectly by extraglomerular disease process [1]. These extraglomerular disease processes may be neoplasm, toxic reactions to drugs, hypersensitivity, and idiosyncratic reaction [1]. Rifampicin is one of the commonly used standard antituberculosis drugs. However, it has been described to have some nephrotoxic adverse effects [2–6]. The most common type of rifampicin-induced nephrotoxicity is acute renal failure with acute tubular necrosis. Other types of nephrotoxicity are acute interstitial nephritis, light chain proteinuria, and rapidly progressive glomerulonephritis [3]. We herein report a case of rifampicin-associated secondary minimal change disease. Our case patient developed nephrotic syndrome after the start of rifampicin therapy for pulmonary tuberculosis, and there was no associated acute renal failure, acute interstitial nephritis, or acute tubular necrosis. After discontinuation of rifampicin, the patient had complete remission of nephrotic syndrome.

## 2. Case Presentation

A 26-year-old male was admitted to the hospital with a 2-week history of cough with expectoration and high-grade fever. He had a history of loss of appetite for ten days. On admission, his blood pressure was 116/76 mm Hg and his body temperature was 37.8°C. Rest of the physical examination was unremarkable, and urine analysis did not show any abnormal findings. The patient's laboratory profile was as follows: hemoglobin: 10.6 g/dL; total leukocyte count: 4,100/mm^3^; platelet count: 2.6 × 10^5^/mm^3^; erythrocyte sedimentation rate (ESR): 58 mm/hr; serum creatinine: 0.86 mg/dL; sodium: 138 mEq/L; potassium: 3.7 mEq/L; and serum albumin: 4.2 g/dL. The chest X-ray showed dense homogenous opacity in right upper zone area of lung. PPD (purified protein derivative of tuberculin) skin test showed a positive reaction, and sputum smear for acid-fast bacilli was found to be positive. On the basis of clinical symptoms, high ESR, positive PPD skin test, positive sputum smear for acid-fast bacilli, and chest X-ray findings, the diagnosis of pulmonary tuberculosis was made. The antituberculosis treatment was started with rifampicin 450 mg/day, isoniazid 300 mg/day, ethambutol hydrochloride 800 mg/day, and pyrazinamide 1000 mg/day.

**Figure 1:**
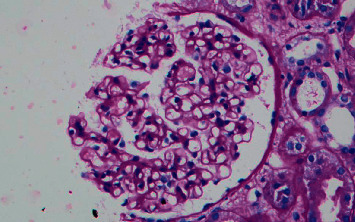
Kidney biopsy specimen on light microscopy showing normal appearing glomerulus without any proliferation or capillary wall thickening (PAS 40x).

**Figure 2:**
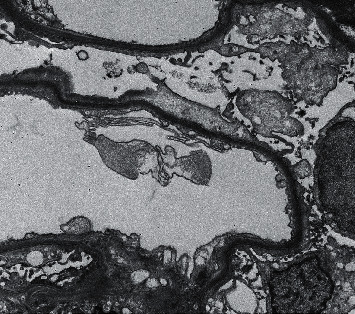
Kidney biopsy specimen with electron microscopy showing diffuse effacement of visceral epithelial cell foot processes (TEM 2000x).

After one month of daily treatment, the patient became sputum smear negative for acid-fast bacilli, but he developed sudden onset swelling whole over the body. The patient's laboratory profile at that time was as follows: hemoglobin: 12.9 g/dL; total leukocyte count: 9,700/mm^3^; platelet count: 2.6 × 10^5^/mm^3^; urinary protein: 3+; urinary sugar: 0; urine microscopy—white blood cell count: 2-3/high-power field; red blood cell count: 0-1/high-power field; urinary pH: 6.2; urinary albumin: 3+; serum albumin: 2.7 g/dL; serum sodium: 136.4 mEq/L; serum potassium: 4.4 mEq/L; blood urea: 36 mg/dL; serum creatinine: 0.82 mg/dL; serum cholesterol: 296 mg/dl; serum glutamic oxaloacetic transaminase (SGOT): 32 U/L; serum glutamic pyruvic transaminase (SGPT): 36 U/L; serum bilirubin total: 0.9 mg/dL; C3: 106.0 mg/dL (normal range: 90–180); C4: 18 mg/dL (normal range: 10–40); serum antinuclear antibody: negative; serum antistreptolysin O titer (ASO titer): <110 IU/mL; cytoplasmic antineutrophil cytoplasmic antibody: negative; perinuclear antineutrophil cytoplasmic antibody: negative; HIV I and II: negative; HBsAg: negative; and anti-HCV: negative. A 24-hour urinary protein value was 10.8 grams/day. Ultrasonography abdomen showed bilateral normal size kidneys with normal echogenicity. A renal biopsy showed nonproliferative glomerulopathy (22 glomeruli) (Figure 1). Tubular atrophy involved less than 10% of the sampled cortex. Tubules showed focally prominent cytoplasmic vacuolar changes, and the arteries sampled appeared unremarkable. Direct immunofluorescence did not show significant glomerular immune deposits. Renal electron microscopy showed diffuse effacement of visceral epithelial cell foot processes (Figure 2). Rifampicin-induced secondary minimal change disease was suspected, and the culprit drug rifampicin was stopped immediately. The other antitubercular drugs were continued with the addition of levofloxacin 500 mg/day. The proteinuria started to decline, and 24-hour urinary protein was 1.2 grams/day after two weeks of stopping of rifampicin. After 30 days of cessation of rifampicin, proteinuria was undetectable in 24-hour urinary samples, and serum albumin and serum cholesterol were found to be normal. The pulmonary tuberculosis was well managed by isoniazid, ethambutol hydrochloride, and levofloxacin, and there was no recurrence of proteinuria.

## 3. Discussion

Various extraglomerular disease processes have been associated with drug-induced secondary minimal change disease (MCD). In the majority of cases, preferably a hypersensitivity reaction appears to be involved, and in some cases, there is a direct toxic effect (e.g., lithium, interferon, and daunomycin) over glomerular capillaries [1]. Nonsteroidal anti-inflammatory drugs (NSAIDs) can cause nephrotic proteinuria and may be associated with glomerular lesions similar to primary minimal change disease. In the majority of NSAID-induced MCD cases, there is an accompanying acute interstitial nephritis specified by the invasion of polyclonal T and B cells [1]. Clinically, NSAID-associated secondary MCD usually presents as acute renal failure. Our case patient developed sudden onset nephrotic proteinuria after one month of continuous use of rifampicin. The glomeruli did not show any immunoglobulins and complement deposition on immunofluorescence examination. Electron microscopy did not show any presence of glomerular immune complexes and thickening of the glomerular basement membrane, promoting the diagnosis of minimal change nephrotic syndrome.

According to De Vriese and coworkers, the most common clinical presentation of rifampicin-associated nephrotoxicity was acute renal failure, and it was due to acute tubular necrosis in the majority of cases, while acute interstitial nephritis was found in some cases [3]. They reported that rifampicin-induced acute renal failure occurred commonly in those patients who had intermittent or interrupted treatment. Our case patient was on continuous rifampicin treatment, and there was no concomitant acute renal failure and acute interstitial nephritis. Richard J. Glassock stated that there is a possibility of development of peculiar changes in permselectivity and morphology of glomerular capillaries in case of secondary MCD, and these changes are similar to primary MCD [1]. Re-exposure to the drug may cause recrudescence, and it is suggestive of a functioning cell-mediated hypersensitivity reaction [1]. Disappearance of proteinuria and full recovery are usually associated with withdrawal of the offending drug. The activated inflammatory or immune cells release permselectivity promoting factors, and this may be the reason for the association between MCD and drug exposure [1]. We thoroughly scrutinized his medical history including medication use. He had no other relevant medical history. We observed that he developed heavy proteinuria only after the start of antitubercular treatment. Favourably, his proteinuria improved after discontinuation of rifampicin. However, it is not yet proven, but there is a possibility that genetic susceptibility can play a role in the occurrence of MCD after drug exposure [1]. There was no such type of family history in our case patient. Yoshioka K and coworkers reported rapidly progressive glomerulonephritis due to rifampicin therapy [6]. Antirifampicin antibody was detected in a patient with crescentic glomerulonephritis by Ogata and coworkers, and they suggested a relationship between renal histology and antibody [3]. De Vriese and coworkers explained that antirifampicin antibody detection was restricted to only those patients who had acute renal failure after rifampicin therapy [3].

Neugarten and coworkers reported the first case of rifampicin-associated nephrotic syndrome with acute interstitial nephritis [7]. There was no immunoglobulin and complement deposition in the glomeruli. The electron microscopy showed dense deposits in the mesangial matrix, subendothelial, and paramesangial areas. They postulated the hypothesis that there was a development of humoral and cell-mediated immune responses to rifampicin treatment [7]. Tada and coworkers also reported rifampicin-associated secondary minimal change disease [3]. They hypothesized that rifampicin-induced endothelial injury was responsible for the development of nephrotic syndrome. Dong Hyuk Park and coworkers reported that rifampicin-induced minimal change disease was improved after cessation of rifampicin without the need of steroid therapy [8]. There were no immune complex or electron dense deposits in the glomeruli, and it was not suggestive of a humoral immune mechanism. They hypothesized that rifampicin-induced MCD was due to a direct toxic effect of the drug [8]. Jee-Seon Kim and coworkers reported rifampicin-induced minimal change disease with acute renal failure in a patient with latent tuberculosis [9]. This patient required temporary dialysis and recovered after discontinuation of rifampicin with steroid therapy [9]. However, our case patient did not demonstrate any renal failure, interstitial edema, or inflammatory cell infiltration or fibrosis, and the patient recovered without the need of steroid therapy.

## 4. Conclusion

From the clinical point of view, we should be aware that sometimes apparently looking primary MCD may be a representation of a secondary lesion due to an extraglomerular disease process. Although secondary MCD is a rare side effect of rifampicin, it is advisable for monitoring of proteinuria during follow-up of patients who are on rifampicin therapy.

## Data Availability

The data sets used and/or analyzed during the current case report are available from the corresponding author upon reasonable request.
